# Extending the Reach of the STOMP Initiative to a Residential Nursing Home in Northern Ireland

**DOI:** 10.1192/bjo.2024.437

**Published:** 2024-08-01

**Authors:** Emily Stirling, Michael Doris

**Affiliations:** Belfast Health and Social Care Trust, Belfast, United Kingdom

## Abstract

**Aims:**

STOMP (stopping the over-medication of people with a learning disability, autism, or both) is a national project launched by NHS England in 2016. The objective is to curb the excessive use of psychotropic medication in individuals with a learning disability, autism, or both to manage behaviour that challenges. This means ensuring that medications are prescribed at the lowest effective dose for the shortest duration of time, and aiming to discontinue if appropriate.

We aim to broaden the implementation of the STOMP initiative to a relatively new residential nursing home in Northern Ireland that is home to individuals with learning disabilities and complex care needs. The residents are discussed at monthly MDT meetings attended by psychiatry, positive behaviour support (PBS) practitioners, activities coordinators, and nursing home managers.

**Methods:**

The inclusion criteria for STOMP are 1. Diagnosis of learning disability, autism, or both, 2. Currently taking psychotropic medication primarily for behaviour that challenges and 3. No diagnosis of severe and enduring mental illness. Five patients were eligible for STOMP.

Outpatient letters and medication prescriptions from the time of admission were compared with the most recent outpatient letters and medication prescriptions.

**Results:**

The five residents were on a range of psychotropic medications including antipsychotics, antidepressants, benzodiazepines, and antihistamines. Following STOMP implementation there was a reduction in psychotropic medication for 80% of the residents.

Patient 1: Reduction in antipsychotic from 75% BNF max daily dose to 40%.

Patient 2: Previously on two antipsychotics with combined use of 75% BNF max daily dose – both medications now discontinued.

Patient 3: Reduction in antipsychotic from 69% max daily BNF dose to 50%, PRN antihistamine discontinued.

Patient 4: PRN antipsychotic discontinued from 15% max daily BNF dose, benzodiazepine use reduced by 5%.

Patient 5: Antipsychotic use increased from 25% max daily BNF dose to 33%.

**Conclusion:**

There was a reduction in psychotropic medication in 80% of the residents. This is an encouraging finding and shows that the STOMP initiative can be expanded to include residential nursing homes. Despite relatively limited resources for STOMP implementation in our local service, we have shown that by keeping the STOMP ethos at the centre of our thinking during monthly MDT meetings involving nursing home management, PBS practitioners, psychiatry, and activities coordinators, we can make sustained reductions in psychotropic prescribing.

